# Effects of Parent-Teacher Training on Academic Performance and Parental Anxiety in School-Aged Children With Attention-Deficit/Hyperactivity Disorder: A Cluster Randomized Controlled Trial in Shanghai, China

**DOI:** 10.3389/fpsyg.2021.733450

**Published:** 2021-12-09

**Authors:** Li Shen, Chunxia Wang, Yuan Tian, Jinjin Chen, Yu Wang, Guangjun Yu

**Affiliations:** ^1^Department of Child Health Care, Shanghai Children’s Hospital, Shanghai Jiao Tong University, Shanghai, China; ^2^Clinical Research Unit, Shanghai Jiao Tong University Affiliated Sixth People’s Hospital, Shanghai, China; ^3^Department of Critical Care Medicine, Shanghai Children’s Hospital, Shanghai Jiao Tong University, Shanghai, China

**Keywords:** ADHD, parent-teacher training, academic performance, parental anxiety, randomized controlled trial

## Abstract

Attention-Deficit/Hyperactivity Disorder (ADHD) is the most common chronic neurodevelopmental disorder in childhood, placing a heavy burden on family and society. The treatment of school-aged children with ADHD emphasizes multimodal interventions, but most current research focuses solely on parent training and family functioning. The aim of this study was to examine the effect of parent-teacher training on academic performance and parental anxiety. In an open-label cluster randomized controlled trial from January 2018 to January 2019, 14 primary schools in Shanghai were randomly assigned into the intervention group and the control group, and ADHD screening was conducted for students from grades one to five. Children in both groups received medication as prescribe by their pediatricians. In the intervention group, families and teachers also received parent-teacher training. The training included ADHD behavioral interventions for parents, as well as classroom management skills for teachers. This study screened 9,295 students, 99 children in the control group and 105 children in the intervention group were included in the analysis. The intervention group demonstrated significant improvement in ADHD symptoms and academic performance and decreases in parent stress compared to that in the control group (*P* < 0.05). This training improved the parents’ perception of ADHD knowledge, treatment options, and drug side effects awareness (*P* < 0.05). Our study aims to underscore the suitability of such programs in the local nuances of the Chinese context, show application feasibility to pediatricians and psychiatrists, and provide supporting evidence for their utilization within the country’s health and educational systems.

## Introduction

Attention-defici/hyperactivity disorder (ADHD) is one of the most common chronic neurological diseases in early childhood (Ameringer and Leventhal, 2013). Numerous studies have showed increasing prevalence rates of ADHD, the prevalence in children aged 6–12 years old is reported as the highest: between 9.8 and 13.3% (Willcutt, 2012; Bronsard et al., 2016). In China, the prevalence of ADHD is around 6.26% of the school-aged children (Grandjean and Landrigan, 2014; Wang et al., 2017).

ADHD brings a heavy burden and induces a variety of undesirable effects to children, parent and society. Children with ADHD have poor academic performance, quality of life, and high incidence of conflicts with parents or teachers (Lieshout et al., 2016). Parents of children with ADHD have increased parenting stress compared to parents of children with any other chronic disease, partially due to misconceptions and lack of ADHD knowledge (Zhu et al., 2015).

Currently, evidence-based treatment for school-aged children with ADHD is medical and behavioral treatment, but both therapies have limitations (Osland et al., 2018). Stimulant medication is the pharmacological treatment of choice for managing ADHD, having been endorsed by hundreds of efficacy studies to reduce core symptoms (Getahun et al., 2013; Konstenius et al., 2014). In general, medical treatment continues to be the most widely used, although it also has important limitations. Medical treatment has efficacy for most ADHD children in reducing core symptoms, however, the sustained use of methylphenidate or atomoxetine may induce drug side effects such as loss of appetite, sleep problems, and mood fluctuations (Upadhyaya et al., 2015; Lo et al., 2016).

Despite shown efficacy, quite often medical treatment suffers from poor compliance (Weisman et al., 2018), due to a general lack in parents of disease-related knowledge and recognition of the importance of medicine in the treatment landscape of ADHD. Moreover, studies have shown that medical dropout rates ranging from 13 to 64% have been observed, particularly in immediate action stimulants (Adler and Nierenberg, 2010). These shortcomings justify the implementation of psychosocial interventions that have been empirically validated as training in behavior management techniques for parents and teachers (Jessica et al., 2019). Psychosocial interventions have been considered to be well-founded and evidence-based treatments for managing the main symptoms of ADHD and have a positive effect on some executive function (Miranda et al., 2013). Previous studies have found that interventions in the school environment can reduce core symptoms of ADHD, cognitive difficulties, disruptive, and aggressive behaviors, while parental interventions can reduce family stress and improve parenting skills (Dupaul and Stoner, 2014; Amado et al., 2016; Reale et al., 2017). However, most behavioral interventions are not systematic, very few studies have included interventions for both parents and teachers.

Parent-teacher training linked family and school systems to provide support for school-aged children with ADHD (Mautone et al., 2011). This training aims to promote home and school well-functioning, strengthen the parent-child relationship, improve parents’ behavior management skills, and promote family-school collaboration through the use of components of behavioral consultation, homework interventions and daily report cards. In one of the first studies by the MTA collaborative Group explored the effects of parent-teacher training, there was no significant differences between the combined and medicine-only group in terms of the core symptoms of ADHD possibly as a result of the changes in the medication procedure (MTA Cooperative Group, 2004; Scruggs and Mastropieri, 2009). Also, fewer studies have focused on the effects of parent-teacher training on academic performance and parental anxiety in school-aged children. The effect of parent-teacher training for children with ADHD is still controversial due to the small sample size, short follow-up time and non-randomized design. Additionally, most studies have included pre-school children, not school-aged children (Epstein et al., 2016; Watzke et al., 2017).

Our study is a cluster randomized controlled trial (RCT) to investigate the effect and acceptability of parent-teacher training, and to the best of our knowledge, it is one of the earliest attempts to evaluate the effectiveness and acceptability of pediatrician-guided parent-teacher training in China. This study aims to underscore the suitability of such programs in the local nuances of the Chinese context, show application feasibility to pediatricians and psychiatrists, and provide supporting evidence for their utilization within the country’s health and educational systems.

### Aims

We aimed to explore whether the pediatrician-guided parent-teacher training could deliver a benefit for children with ADHD, compared with only received medication treatment. We hypothesis that compared with the children in the control group, the children in the intervention group would reduce ADHD symptoms, parental stress and improve academic performance after the intervention.

## Materials and Methods

### Study Design

This study was a cluster RCT to investigate the effect and acceptability of the parent-teacher training after 4 and 10 month. It consists of three parts: ADHD screening, parent-teacher training implementation, and evaluation the effect and acceptability of the parent-teacher training.

### Setting and Population

The RCT was carried out at 14 primary schools (children aged 6–11 years) located in Jiading District, Shanghai, China. A meeting convened by professional pediatricians aims to give the teachers and the parents a better understanding of the goal of the research and the specific pattern of intervention. Parents and teachers who agreed to participate in the program must sign an informed consent. This study was conducted from January 2018 to December 2018. Parents of children who screened positive were notified by phone or e-mail to take their children to Shanghai Children’s Hospital for specialized examination. Eligible patients were included after obtaining written informed consent.

### Study Participants

Inclusion criteria: (1) Meet the diagnosis of ADHD according to Diagnostic and Statistical Manual of Mental Disorders (DSM-5) (American Psychiatric Association, 2013). (2) Between 6–12 years old. (3) Parents agree to use methylphenidate or tomoxetine for treatment. (4) Parents can read and write the Chinese language. (5) Parents signed the informed consent.

Exclusion criteria: (1) Intellectual disability (IQ < 70). (2) excluded autistic spectrum disorder (ASD), epilepsy, schizophrenia, cerebral palsy and other nervous system diseases and mental disorders. (3) Excluded children with severe heart, brain, kidney, and other organ dysfunction. (4) Children with hearing and visual impairment.

### Randomization and Blinding

The randomization sequence was created by a third-party statistician using SAS statistical software version 9.1 (SAS Institute Inc., Cary, NC, United States). A total of 14 schools were included in this study and were randomly divided into the intervention group (seven schools) and the control group (seven schools). Parents, teachers, and pediatricians were aware of the allocated arm. A senior statistician who was blinded to the group allocation carried out the primary and secondary analysis.

### Interventions

This study provided a multimodal treatment for teachers and parents in the intervention group, which integrates family, school, and medical department to address the aims of a holistic ADHD management strategy. The parent-teacher training herein was 16-week training for parents and teachers, the three components: teacher training, family training, and ADHD knowledge improvement; and applied medical treatment were described below. Details describing the treatment components were provided in Supplementary Table 1.

### Medical Treatment

Children in both the intervention and control groups were treated with medication. All children started stimulant medication as prescribed by their pediatricians, referring to the clinical practice guidelines for ADHD children published by the American Academy of Pediatrics (2000).

### Teacher Training

The intervention was implemented by hospital pediatricians targeting to improve the knowledge of ADHD, behavioral intervention and teachers’ classroom management. Hospital pediatricians conducted 1 group meeting with participating school teachers (30-min introduction session, 1-h classroom behavior management, and 30-min case introduction) and 1 school-based consultation with parents and school teachers (30 min communication between parents and teachers about the performance of children). Teachers completed the daily behavior report card and homework plan (Pfiffner et al., 2018).

### Parent Training

The intervention began with an overview of the study and behavioral intervention for ADHD children conducted by hospital pediatricians. Hospital pediatricians conducted 2 group parent trainings sessions (30-min introduction session, 2-h ADHD knowledge course, 1-h family behavior management course, and 1-h case introduction) and 2 individualized family therapy sessions (1-h communication between hospital pediatricians and parent and 1-h parent-children interaction). The training included the use of commands, rewards, discipline, and stress management. There were two conferences calls between the hospital pediatricians and the parents (approximately for 10 min) to monitor the children’s treatment and to improve the intervention if needed.

### ADHD Knowledge Promotion

This study established a WeChat messaging platform-based (Tencent Holdings Limited, Shenzhen, China) official account, which was followed by the parents. ADHD information was published to parents and teachers with the goal to inform about ADHD and gain disease-related knowledge through pictures, texts, and videos. In addition, this study distributed two training manuals to introduce the symptoms, causes, diagnosis, treatment, and intervention of ADHD to the children in the intervention group (Fleming et al., 2017).

### Intervention Delivery

This training was conducted by multi-disciplinary research team whose participants are hospital pediatricians, psychologist, statistician, postgraduates, and teachers in 14 primary schools in Jiading District. All teacher and parent trainings were held in the classrooms of primary schools and the intervention was conducted by the hospital pediatricians. Ninety five percentage teachers participated in teacher training, and 80% parents participated in family training. This study contacted with parents by telephone every month and inquired about treatment status, medication status, and drug side effects.

### Ethics

This study has been reviewed and approved by the Research Ethics Committee of Shanghai Children’s Hospital (2018R003) and registered at the Chinese Clinical Trial Registry^1^ as ChiCTR1800014945. All participants signed the informed consent.

## Measures

### Screening

Researchers use Conners Parent Symptom Questionnaire (PSQ) and Conners Teacher Rating Scale (TRS) to assess the symptoms of ADHD in primary school children. There are 48 items in PSQ, including six factors including conduct problems, learning problems, psychosomatic, hyperactivity-impulsivity, anxiety, and ADHD index (Conners et al., 1998). The higher the scores, the higher the probability of having ADHD, if the ADHD index is ≥ 1.5, indicating that the child may have ADHD. TRS is a scale used by teachers to assess behavioral problems in children. It contains 28 items, including five factors of conduct, hyperactivity, inattention, and ADHD index. The higher the scores, the higher the probability of having ADHD, if ADHD index ≥ 1.5, indicating that the child may have ADHD.

### Primary Outcome Measures

Swanson Nolan and Pelham’s fourth scale (SNAP-IV) contains 26 items, including three factors of inattention, hyperactivity, and impulsivity. The higher the score, the more severe the ADHD symptoms. Researchers use it to assess the severity of core symptoms of ADHD. The whole consistency Cronbach α = 0.94, the subscales for attention deficit, hyperactivity – impulsivity, and oppositional disobedience were 0.90, 0.79, and 0.89, respectively.

### Secondary Outcome Measures

The secondary outcomes consisted of academic performance and parent stress, all questionnaires were measured at baseline (t1), the end of the treatment (t2), and 10-month (t3) (see Supplementary Table 2).

#### Academic Performance Questionnaire

Academic Performance Questionnaire (APQ) was developed based on the Olivero Bruni’s Teacher school achievement form and school-teacher can APQ to assess students’ academic performance. The questionnaire consists of 15 items using 5-point scale ranging from 1 to 5, with higher scores indicating better performance and four subscales (attention, study interest, school achievement, and executive ability). The internal consistency Cronbach α = 0.962 (Zhang et al., 2015).

#### Parenting Stress Index

Parenting Stress Index (PSI) is used to assess the difficulties, anxiety, nervousness, and the level of parenting stress which aims to identify various difficulties in children’s growth and development (Haskett et al., 2017). It consists of 36 items using a 5-point scale ranging from 1 to 5 and three subscales (parent stress, parent-child dysfunctional interaction, and difficult child). The internal consistency Cronbach α = 0.88.

#### Treatment Acceptability Questionnaire

The Treatment Acceptability Questionnaire (TAQ) scale were rated on a 6-point Likert-type scale, ranging from 1 (strongly disagree) to 6 (strongly agree) with higher scores indicating the greater acceptability. TAQ is 8-item measure and is widely used in the treatment acceptability assessment of ADHD children (Krain et al., 2005).

### Demographic Variables

This study collected the child’s gender, child’s age, child’s grade, primary caregivers, socioeconomic status, family structure, parents’ age, parents’ education degree, and child’s ADHD medication status.

### Power and Sample Size

We used PASS 16 software (NCSS, the United States) to calculate the sample size. The sample size was based on the primary outcome of this study; the SNAP-IV scores at 10 months between the intervention group and the control group. In this study, we assumed the difference of mean scores in the intervention group and the control group was 0.2 with the standard deviation (SD) 0.48 (Power et al., 2012). Since this study is an exploratory test, a two-sided test was used. In order to defect 80% of the power of this difference when a two-sided alpha = 0.05 is applied, each group size of 88 (total sample size *n* = 176) is needed. In order to adjust the drop out and loss of 10%, 194 participants will be needed.

### Statistical Analyses

In this study, 105 children were eventually in the intervention group and 99 children were included in the control group. Multiple imputation methods were used to replace the missing data in the primary efficacy analysis to reduce potential confounding bias. The baseline variables of the two groups were compared by analysis of a *t*-test for continuous variables and Chi-square tests for categorical variables.

Comparisons between the intervention group and the control group for the continuous outcome variables were performed with the use of linear mixed effect model to repeated measures. We had planned to use a linear mixed-effect model for the primary and secondary outcomes analysis. The model included group, time, the interaction of group with time as fixed effects and child-specific random intercepts, child age and mother age as covariates.

All data were performed with SAS software version 9.2 (SAS Institute, Cary, NC, United States) and R version 4.0.2^2^ to evaluate intervention effects on the primary outcomes and secondary outcomes.

## Results

### Baseline

**FIGURE 1:**
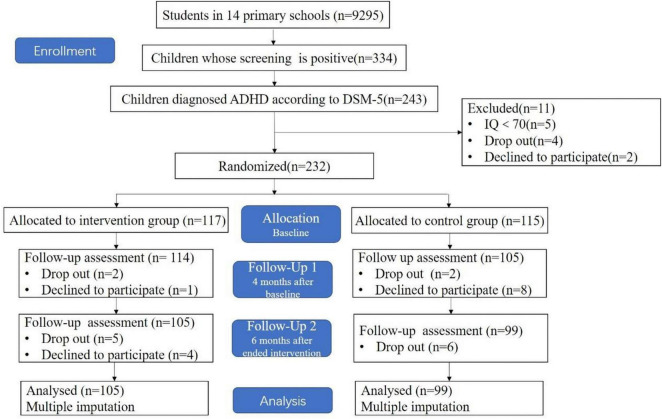
Flow diagram of participant enrollment and trial randomization.

A total of 9,295 children completed the screening questionnaire, 334 (3.59%) were positive for the screening. Of those 334 children screening positive children, 243 (72.75%) were diagnosed ADHD. However, 21 children were excluded because 5 children have developmental delay, 4 students drop out of school, and 12 declined to participate this study. Information on the flow diagram of participant enrollment in this study is shown in Figure 1. A total of 204 children were included in the analysis. There were no serious adverse events and adverse events reported.

Table 1 shows the demographics and treatment status of the 99 children in the control group and 105 children in the intervention group. There were no statistically significant between the intervention group and the control group except the child age and mother age (*P* > 0.05). As shown in Table 1, there were 85 boys (85.9%) and the mean age was 8.39 in the intervention group. There were 90 boys (85.7%) and the mean age was 7.71 in the control group.

**TABLE 1:** Demographic characteristics of intervention group and control group.

Variable	Control	Intervention	χ^2^/t (*P*)
	(*n* = 99)	(*n* = 105)	
Child age, x±s	8.39 ± 1.38	7.71 ± 1.22	3.745 (0.000)
Gender, *n* (%)			0.001 (0.976)
Boy	85 (85.9%)	90 (85.7%)	
Girl	14 (14.1%)	15 (14.3%)	
Grade, *n* (%)			2.310(0.129)
∼3 grade	34 (34.3%)	47 (44.8%)	
3 grade∼	65 (65.7%)	58 (55.2%)	
ADHD subtype			0.032(0.984)
Inattention	40 (40.4%)	42 (40.0%)	
Hyperactive-impulsive	29 (29.3%)	30 (28.6%)	
Combined	30 (30.3%)	33 (31.4%)	
Comorbidit			0.588(0.989)
Tourette’s disorder	17 (17.2%)	18 (17.1%)	
Oppositional defiance	8 (8.1%)	11 (10.5%)	
Conduct disorder	10 (10.1%)	9 (8.6%)	
Mood disorders	8 (8.1%)	7 (6.7%)	
AS	2 (2.0%)	2 (1.9%)	
Primary Caregiver, *n* (%)			0.812 (0.847)
Father	15 (15.2%)	18 (17.1%)	
Mother	67 (67.7%)	72 (68.6%)	
Grandparents	15 (15.1%)	12 (11.4%)	
Other	2 (2.0%)	3 (2.9%)	
Father age, x±s	36.96 ± 5.02	36.50 ± 5.37	0.637 (0.525)
Mother age, x±s	35.07 ± 4.14	33.76 ± 4.07	2.277 (0.024)
Father education, *n* (%)			1.410 (0.494)
∼Junior high school	23 (23.2%)	29 (27.6%)	
High school∼ College	55 (55.6%)	60 (57.1%)	
College∼	21 (21.2%)	16 (15.2%)	
Mother education, *n* (%)			5.441 (0.066)
∼Junior high school	27 (27.3%)	35 (33.3%)	
High school∼ College	46 (46.5%)	56 (53.3%)	
College∼	26 (26.3%)	14 (13.3%)	
Family structure, *n* (%)			1.644 (0.649)
Stem family	46 (46.5%)	43 (41.0%)	
Core family	48 (48.5%)	53 (50.5%)	
Single parent family	2 (2.0%)	5 (4.8%)	
Intergenerational family	3 (3.0%)	4 (3.8%)	
Family income/yuan, *n* (%)			3.194 (0.526)
∼5000	7 (7.1%)	13 (12.4%)	
5000∼10000	37 (37.4%)	38 (36.2%)	
10000∼15000	23 (23.2%)	29 (27.6%)	
15000∼20000	19 (19.2%)	15 (14.3%)	
20000∼	13 (13.1%)	10 (9.5%)	

### Treatment Acceptability

In this study, 64.8% of the parents in the intervention group hold the opinion that this treatment would help their children. Supplementary Table 3 showed the mean and SDs of the eight items scores for the TAQ of the intervention group.

### Overall Intervention Effect

**FIGURE 2:**
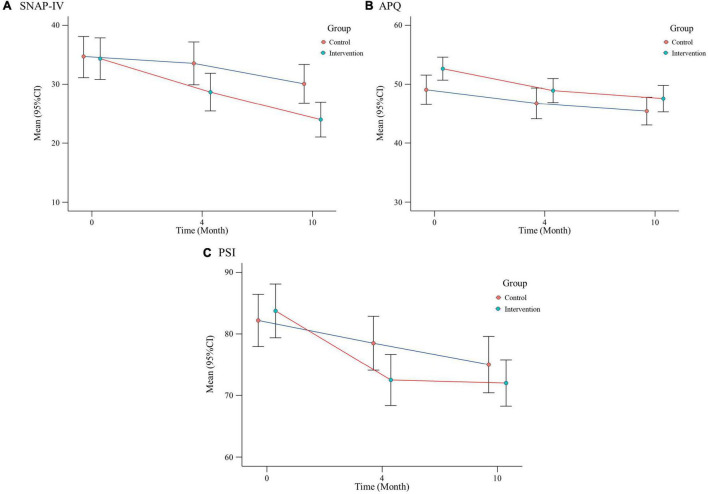
Effects of interventions over time: changes in SNAP-IV, APQ, and PSI in two groups of participants at different visit points.

We first analyzed the mean and 95% CI for the outcome variables at different time point and change from baseline between the two groups (see Table 2). SNAP-IV scores decreased at different time points in both the intervention and control group (see Figure 2). From baseline to 10 months, mean scores of SNAP-IV decreased from 34.34 (95% CI:30.81–37.87) to 23.98 (95%CI:21.02–26.94) in the intervention group and from 34.72 (95% CI:31.33–38.10) to 30.07 (95% CI:26.78–33.36) in the control group. Changes over time are shown in Figure 2. The Figure 2 showed that the SNAP-IV scores, APQ scores, and PSI scores were improved in both the intervention group and the control at post-treatment and follow-up.

**TABLE 2:** Mean values and changes from baseline for outcomes.

	Mean values (mean, 95% CI)		Change from baseline (mean, 95% CI)	
	Control	Intervention	Control	Intervention
**SNAP-IV**				
Baseline	34.72 (31.33–38.10)	34.34 (30.81–37.87)		
At 4 month	33.54 (29.90–37.17)	28.65 (25.45–31.86)	−1.18 (−4.47, 2.11)	−5.69 (−10.26, −1.11)
At 10 month	30.07 (26.78–33.36)	23.98 (21.02–26.94)	−4.65 (−8.28, −1.02)	−10.36 (−13.84, −6.88)
**APQ**				
Baseline	49.05 (46.55–51.55)	52.64 (50.68–54.60)		
At 4 month	46.74 (44.11–49.36)	48.90 (46.85–50.96)	−2.31 (−4.11, −0.52)	−3.73 (−6.26, −1.21)
At 10 month	45.42 (43.06–47.79)	47.55 (45.29–49.80)	−3.63 (−6.56, −0.70)	−5.09 (−6.87, −3.31)
**PSI**				
Baseline	82.18 (77.94–86.43)	83.74 (79.37–88.11)		
At 4 month	78.48 (74.10–82.87)	72.49 (68.33–76.64)	−3.70 (−7.49, 0.10)	−11.26 (−16.17, −6.34)
At 10 month	75.00 (70.41–79.59)	71.99 (68.23–75.76)	−7.18 (−12.82, −1.54)	−11.75 (−15.83, −7.68)

The mixed effect models revealed that the greater improvements in ADHD symptoms (*F* = 8.27, *P* = 0.005), academic performance (*F* = 11.05, *P* = 0.001) and parent stress (*F* = 4.30, *P* = 0.042) in the intervention group as compared to the control group. The mixed model showed significant difference in the ADHD performance, academic performance, and parent stress over time (*P* < 0.05). There was no significant time×group effect on all outcome variables (*P* > 0.05) (see Table 3).

**TABLE 3:** Results of mixed effect models of the outcome measures between the intervention group and the control group.

		*F*(*P*)	
		
	Time	Group	Time×group
SNAP-IV	12.48 (0.000)	8.27 (0.005)	2.01 (0.139)
APQ	9.54 (0.000)	11.05 (0.001)	0.33 (0.718)
PSI	14.11 (0.000)	4.30 (0.042)	2.05 (0.132)

### Parents’ Knowledge, Treatment Options, and Drug Side Effect Toward Treatment Before and After Training

There were statistically significant differences in ADHD knowledge, treatment options, and drug side effects between baseline and 4-month follow-up (*P* < 0.05). Before training, there were only 28.1% believed that ADHD was a neurological disorder, 14.9% believed medication was the first-line treatment and 13.2% parents drug effects answered correctly. While after training, 44.7% believed that ADHD was a neurological disorder, 27.2% believed medication was the first-line treatment and 42.1% parents answered drug effects correctly (see Table 4).

**TABLE 4:** Parents’ knowledge, treatment options, and drug effect toward treatment before and after training (intervention group).

		Before (*n*, %)	After (*n*, %)	χ^2^	*P*
Knowledge	Is a disorder	32 (28.1)	51 (44.7)	7.661	0.022
	Not a disorder	64 (56.1)	45 (39.5)		
	Not sure	18 (15.8)	18 (15.8)		
Treatment options	Medicine	17 (14.9)	31 (27.2)	13.52	0.004
	Behavioral intervention	56 (49.1)	48 (42.1)		
	Education	20 (17.5)	6 (5.3)		
	Not sure	21 (18.4)	29 (25.4)		
Drug effect	Correct answer	15 (13.2)	48 (42.1)	23.89	0.000
	Wrong answer	99 (86.8)	66 (57.9)		

## Discussion

This study provided a multimodal treatment for patients and teachers in the intervention group, which integrates family, school, and health care providers’ efforts to address the aims of a holistic ADHD management strategy. This study provides evidence for the effectiveness of parent-teacher training for school-aged children with ADHD. Parent-teacher training has a significant impact on the family involvement in education, academic performance, and parental stress. ADHD treatment paradigm consists of long-term standardized medical treatment and non-medical treatments. The superiority of parent-teacher training was verified and all outcome variables were changed significantly over time. This study referred to previous studies demonstrating effective behavioral intervention in children with ADHD (Goossens et al., 2016). In addition, this study showed that parent-teacher training is an acceptable treatment.

This study found that during the intervention period, children in the intervention group showed a gradual reduction in their core symptoms and an improvement in their academic performance. The American Academy of Pediatrics emphasizes that behavioral therapy combined with pharmacotherapy is effective, and behavioral interventions for parents and classroom behavioral interventions for teachers are very effective in changing the core symptoms of children with ADHD (American Academy of Pediatrics. Committee on Public Education, 2001). ADHD children often had learning dysfunction, including poorer academic performance, classroom disorganization, dropout, and disciplinary behavior. Our findings are in the line with previous studies, teacher training was shown to be effective in reducing the core symptoms of ADHD, difficulties in cognition and disruptive behaviors (Betty et al., 2017). Previous studies have found that poor home environments were associated with poorer academic performance (Polderman et al., 2010). The study showed that it was effective in increasing academic productivity, social competence and rule compliance (Charach et al., 2013). This study enhanced parents’ and teachers’ ADHD knowledge, classroom management skills, and family parenting skills to help children improve their academic performance (Loo et al., 2016).

Stress within a family has wide implication of familial well-being; for example, socio-ecological theories suggest that stress on one family member may influence any other family members and cause disputes and disharmony (Sola et al., 2016). Previous studies have shown parent training do not only change children’s behavior; in some cases, they can also improve family functions and reduce parental stress (Ciesielski et al., 2020). In clinical practice, strengthening disease-related education for parents can improve parents’ perception and knowledge of ADHD, help parents acquire parental skills, gain social support, enhance parent-child interaction, and reduce the stress of caring for children with ADHD (Schuck et al., 2018). This study improved parents’ knowledge of ADHD disease, treatment options and drug side effect. For ADHD children, parent beliefs and attitudes toward ADHD are of critical importance, parents’ belief about ADHD medication outweigh evidence of the real benefits and risk (Veloso et al., 2019). The previous study of the research team found that a significant correlation acceptance of pharmacotherapeutic, and parent-teacher training could increase the medication adherence (Zhang et al., 2020). Multimodal therapies are beneficial because they can help providers directly solve obstacles in multiple fields through parent-teacher collaboration, and increase the participation of families and schools in children’s academic life (Sciutto, 2015).

The results of this study provide support for parent-teacher training. First, this study is the first to demonstrate that a parent-teacher training in ADHD children can decrease the parent stress. Second, unlike most previous studies, this study investigated the differential effectiveness of medical treatment and combined intervention. Most guidelines recommend that medication therapies are the primary treatment for school aged children with ADHD, this study explored the additional effects of Parent-teacher training (Thapar and Cooper, 2016). All teacher and parent trainings were carried out at school. Third, this study found that the combining medicine and parent-teacher intervention has a significant effect on the school-aged children with ADHD. The multimodal intervention of children provides a theoretical basis, further indicating that the ADHD disease is related to multiple mechanisms such as psychological and social factors. Researchers should focus on individualized courses for children of different grades, and how to maintain the effect of intervention.

## Conclusion

Our study is a cluster RCT, session-based training for both teachers and parents and investigate the effect and acceptability of the parent-teacher training after 4 and 10 months after enrollment. The findings from our study indicate that parent-teacher training improves children’s core symptoms and academic performance, and reduces parental anxiety. While at the same time, the intervention improves parental attitudes and perceptions of ADHD.

## Strength and Limitations

Our study is a cluster RCT to investigate the effect and acceptability of pediatrician conducted parent-teacher training and to the best of our knowledge, it is one of the earliest attempts to evaluate the effectiveness and acceptability of parent-teacher training in China. Compared with previous studies, this study has a larger sample size and longer follow-up time.

There are several limitations in this study. Some children who screened positive refused to participate in the study, so the prevalence of ADHD in this study was about 2.5% in this study which is lower than the prevalence reported in the literature. We have not explored the role of possible symptoms of ADHD of the parents in the treatment of their children (Sanchez et al., 2019). Also, this study did not explore the possible factors that may condition the response to treatment, as the MTA study has shown (Swanson et al., 2001). Finally, the ADHD symptoms were only assessed by parents, and teachers’ assessment results were not collected.

## Data Availability Statement

The datasets generated for this study are available on request to the corresponding author.

## Ethics Statement

The studies involving human participants were reviewed and approved by the Research Ethics Committee of Shanghai Children’s Hospital (2018R003). Written informed consent to participate in this study was provided by the participants’ legal guardian/next of kin.

## Author Contributions

LS and YW designed this research. JC and YT coordinated the implementation of this research. LS wrote the manuscript. LS and CW contributed to the analysis of this research. All authors have read and approved the final manuscript.

## Conflict of Interest

The authors declare that the research was conducted in the absence of any commercial or financial relationships that could be construed as a potential conflict of interest.

## Publisher’s Note

All claims expressed in this article are solely those of the authors and do not necessarily represent those of their affiliated organizations, or those of the publisher, the editors and the reviewers. Any product that may be evaluated in this article, or claim that may be made by its manufacturer, is not guaranteed or endorsed by the publisher.

## Footnotes

1
www.chictr.org.cn

2
http://www.r-project.org
