# Supplementary material for: Effects of Parent-Teacher Training on Academic Performance and Parental Anxiety in School-Aged Children With Attention-Deficit/Hyperactivity Disorder: A Cluster Randomized Controlled Trial in Shanghai, China
Source: Front Psychol. 2021 Dec 9;12:733450. doi: 10.3389/fpsyg.2021.733450 (PMC8695601; doi:10.3389/fpsyg.2021.733450)
Supplement: Supplementary file 1 [file Data_Sheet_1.docx]

**Supplement legends**

**Supplement 1.** Description of family-school intervention

**Supplement 2.** Measurement of endpoint indicators at different time points

**Supplement 3.** Descriptive Statistics for Measures of Treatment Acceptability Questionnaire

**Supplement 1.** Description of family-school intervention

| Components | Sessions | Details |
| --- | --- | --- |
| Teacher training | Introduction session | Research purpose, and type of the interventions |
|  | Classroom behavior management | ADHD manifestation and etiology |
|  |  | Establishing classroom behavior standards |
|  |  | Designing special courses for ADHD children |
|  | Case introduction | Analyzing the behavior of children and teachers in specific cases |
|  |  | Learning to understand ADHD children |
|  | School-based consultations | Ways for teachers to communicate with ADHD parents |
|  |  | Ways for parents to communicate with pediatrician |
|  |  | Communication about the performance of children |
| Parent training | Introduction session | Research purpose, content and family-school interventions |
|  | ADHD knowledge course | ADHD manifestation, etiology, diagnosis and medicine treatment |
|  | Family behavior management course | Stress management skills |
|  |  | Developing children’s learning ability |
|  |  | Improving parent-child communication |
|  |  | Formulating family rules |
|  |  | Using incentives and penalties |
|  | Case introduction | Analyzing the behavior of children and parents in specific cases |
|  | Individualized family therapy session | communication between hospital pediatricians and parent to solve problems together |
|  |  | Parent-child game interaction to promote parent-child relationship |
| ADHD knowledge promotion | WeChat official account | Disease-related knowledge through pictures, texts, and videos |
|  | Training manuals | Introducing disease knowledge and family management skills |

**Supplement 2.** Measurement of endpoint indicators at different time points

|  | Measure | Informant | T1 | T2 | T3 |
| --- | --- | --- | --- | --- | --- |
| Screening | Conners’ Parent Symptom Questionnaire (PSQ) | Parent | √ |  |  |
|  | Conners’ Teacher Rating Scale (TRS) | Teacher | √ |  |  |
| Primary outcomes | Chinese Version of Swanson Nolan and Pelham, Version IV Scale (SNAP-IV) | Parent | √ | √ | √ |
| Secondary outcomes | Academic Performance Questionnaire (APQ) | Teacher | √ | √ | √ |
|  | Parent Stress Index (PSI) | Parent | √ | √ | √ |
|  | Treatment Acceptability Questionnaire(TAQ) |  |  | √ |  |

**Supplement 3.** Descriptive Statistics for Measures of Treatment Acceptability Questionnaire

| Item | Agree(%) | mean | SD |
| --- | --- | --- | --- |
| 1. This is an acceptable treatment for my child’s behavior. | 68.6 | 3.96 | 1.47 |
| 2. This treatment should be effective in changing my child’s behavior. | 70.5 | 3.97 | 1.46 |
| 3. My child’s behavior is troublesome enough to justify the use of this treatment. | 66.7 | 3.88 | 1.50 |
| 4. I would be willing to use this treatment with my child. | 68.6 | 3.95 | 1.47 |
| 5. This treatment would be have had side effects for my child. | 65.7 | 4.00 | 1.56 |
| 6. I like this treatment. | 67.6 | 3.92 | 1.52 |
| 7. This treatment is a good way to handle my child’s problem. | 61.0 | 3.86 | 1.54 |
| 8. Overall, this treatment would help my child. | 64.8 | 3.93 | 1.51 |
